# Intraocular vascular analysis using optical coherence tomography angiography in patients with vascular paralytic strabismus

**DOI:** 10.1371/journal.pone.0272524

**Published:** 2022-09-13

**Authors:** Donghun Lee

**Affiliations:** Department of Ophthalmology, Daegu Catholic University School of Medicine, Daegu, South Korea; Cairo University Kasr Alainy Faculty of Medicine, EGYPT

## Abstract

**Purpose:**

To investigate changes in peripapillary and macular vessel density (VD) in vascular paralytic strabismus using optical coherence tomography angiography (OCTA).

**Methods:**

Medical records of patients who recovered from monocular vascular paralytic strabismus were retrospectively analyzed. Age, sex, presence of underlying diseases, strabismus type and severity, time to recovery, and visual acuity at diagnosis were evaluated. VD in the optic disc area and macular capillary plexus density were estimated using OCTA. The effect of paralytic strabismus on intraocular VD was investigated by comparing VD between the paralysis and contralateral eyes. To analyze hemodynamic changes, VD changes in the paralysis eye during the attack and recovery were compared.

**Results:**

Thirty-one patients (mean age, 64.1±13.0 years; 21 males, 10 females) were included and mean recovery time was 3.0±1.6 months. The most common paralysis was sixth nerve palsy (54.8%). When comparing OCTA results between the paralysis and contralateral non-paralysis eyes, foveal VD in the superficial capillary plexus (SCP) was significantly lower in the paralysis eye (P = 0.034); however, VD in the optic disc area was not different. In the paralysis eye, foveal VD in the SCP significantly increased after paralysis recovery (P = 0.04). During attack, the maximal deviation angle and severity of duction limitation were significantly related to foveal VD in SCP. The greater the deviation angle and the more severe the eye movement restriction, the lower the foveal VD in SCP.

**Conclusions:**

Transient retinal ischemia of the paralysis eye was observed in a patient with paralytic strabismus, which corresponded to the degree of deviation angle and ocular motor restriction. Ischemic factors, which are the etiology of vascular paralytic strabismus, affect intraocular blood flow.

## Introduction

Vascular paralytic strabismus is defined as acquired paralytic strabismus with vascular comorbidities such as diabetes and hypertension, with no history of trauma or neoplastic cause [1, 2]. Blood supply to the third, fourth, and sixth nerves has multiple sources that feed on the vasa nervorum capillary network, and microvasculopathy induces cranial nerve palsy, resulting in vascular paralytic strabismus. This is the most common cause of paralytic strabismus [1] and 38.0%-80.0% of patients show favorable prognosis, with spontaneous regression of symptoms within several weeks [3–5]. Most previous studies on this disease have focused on the epidemiology, prognosis, and clinical features [1, 3, 6]. Structural analysis is rarely performed because the structural assessment of cranial nerve vascularity in paralytic strabismus is limited.

Meanwhile, since 2014, optical coherence tomography angiography (OCTA), which is advantageous because of its non-invasive and timesaving nature, has been used to detect retinal vasculature in vivo, such as in retinal artery and vein occlusion, diabetic retinopathy, and age-related macular degeneration [7, 8]. Subsequently, the application of OCTA has been expanded, and it has been used to evaluate vessel analysis in various intraocular diseases, such as anterior segment ischemia, acute angle closure, and other optic neuropathies [9–11].

Macular perfusion may change in relation to constant lack of normal visual perception by the deviating eye. Furthermore, it was hypothesized that intraocular blood circulation in vascular paralytic strabismus may change according to the disease course. Although the etiology of vascular paralytic strabismus is micro-ischemia in the cranial nerve that innervates eye movement in patients with systemic vascular comorbidities, but non-existing in the eyeball, intraocular vascular structural analysis can be meaningful in evaluating the disease entity and course. Therefore, this retrospective study aimed to test this hypothesis.

## Materials and methods

Data obtained from patients at Daegu Catholic University Hospital who visited the hospital for acute-onset diplopia between January 2020 and April 2021 were retrospectively analyzed. Patients who had completely recovered from monocular vascular paralytic strabismus and had undergone OCTA measurements at least twice from diagnosis to recovery were enrolled in this study. Only patients who underwent eye examinations, including OCTA measurement within two weeks of symptom onset, were enrolled. The present study was conducted with the approval of the Daegu Catholic University Hospital Institutional Review Board (IRB no. CR-22-002-L), and all procedures adhered to the tenets of the Declaration of Helsinki. Informed consent was obtained verbally from all participants, with the their guardians and residents as witnesses.

The diagnostic criteria for vascular paralytic strabismus were a duction abnormality detected with sudden-onset diplopia that worsened on the affected side and the presence of at least one vascular risk factor, namely, diabetes, hypertension, dyslipidemia, ischemic heart disease, or cerebral vascular disease without a history of trauma. Brain magnetic resonance imaging was also performed for cases of ischemic small vessel lesions or microinfarction. In the case of fourth nerve palsy, a positive response was confirmed using the Park-Bielschowsky three-step test [2]. Patients with a history of orbital surgery or recurrent paralytic strabismus were excluded. Furthermore, to exclude factors that may have affected the changes in intraocular vessel density (VD), patients with retinal diseases such as age-related macular disease, diabetic retinopathy, ischemic retinal vessel occlusion, and optic neuropathies including glaucoma and ischemic optic neuropathy were excluded from the study. Other cases, including congenital paralytic strabismus; thyroid eye disease; myasthenia gravis; orbital wall fracture; and brain lesions such as aneurysms, neoplasms, or midbrain infarcts that can directly cause paralytic strabismus, were also excluded.

All patients underwent comprehensive ophthalmological examinations, including measurement of best-corrected visual acuity and intraocular pressure using a non-contact tonometer, non-cycloplegic refractive error assessment using autorefractive keratometry, fundus examination, and slit-lamp examination. Refractive error results were converted to a spherical equivalent, and the best-corrected visual acuity was converted to the logarithm of minimal angle resolution (logMAR) for statistical analysis.

The deviation angle at distance fixation (6 m) was measured using the prism cover test in the primary position. Prism cover test was performed by placing a prism in front of the deviating eye and covering the opposite normal eye. If both the horizontal and vertical deviations coexisted, then the largest deviation was used for the analysis. The severity of ocular movement limitation was graded from -1 to -4 as described by Scott and Kraft [12]. In cases of third nerve palsy, the most severe limitation of eye movement was evaluated. Complete recovery of paralytic strabismus was defined as subjective diplopia resolution, relief of duction limitation, and decrease in the deviation angle to less than 8 prism diopters (PD) [13].

**Fig 1:**
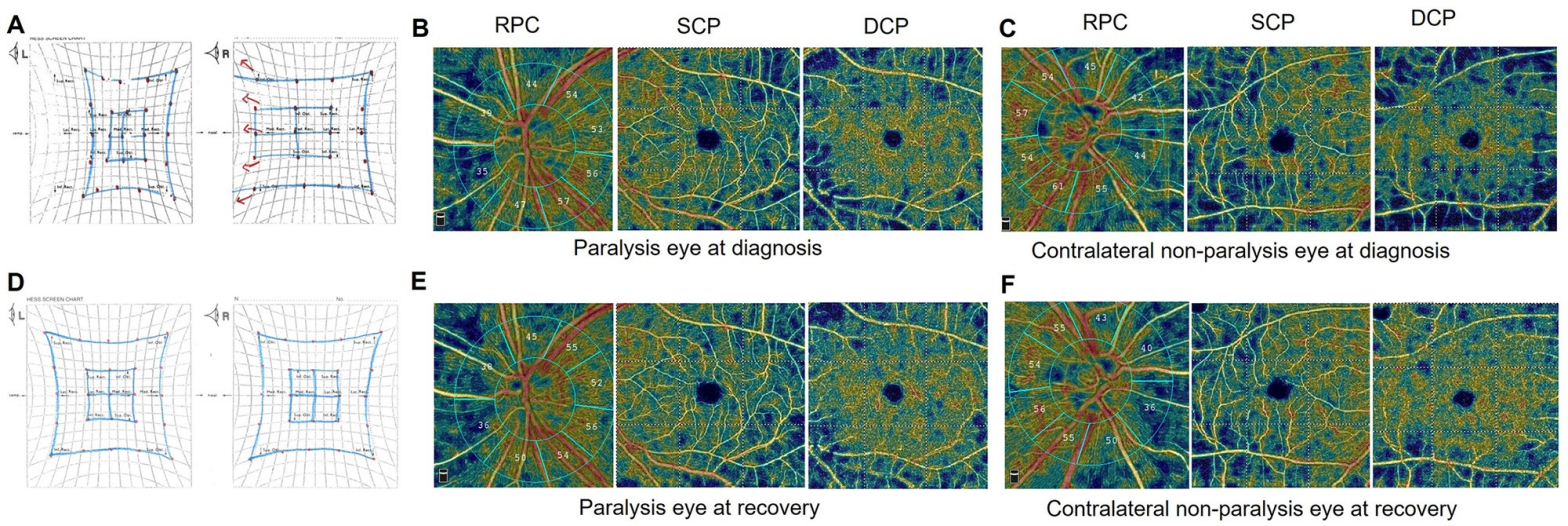
Representative case images of a 66-year-old female patient with left sixth nerve palsy. Optical coherence tomography angiography (OCTA) color-coded scans illustrating the acquisition of various parameters. At the diagnostic point, results of the Hess screen test revealed -2 grade of gaze limitation at lateral gaze of the left eye paralysis eye (A). Vessel densities of radial peripapillary capillary (RPC), superficial capillary plexus (SCP), and deep capillary plexus (DCP) were compared between the paralysis eye (B) and contralateral nonparalysis eye (C). After 3 months, her symptoms of limited eye movement (D) and diplopia had improved. At the recovery phase, vessel densities of RPC, SCP, and DCP in both eyes (E, F) were repeatedly compared.

OCTA data were obtained using the RTVue XR Avanti device (Optovue Inc., Fremont, CA, USA). Microcirculation status in both the optic disc and macula was evaluated, and VD in each intraocular structure was automatically measured using the built-in software program AngioVue^®^. On the optic disc area, a 4.5 × 4.5-mm angio-disc mode was used on the optic disc area, and the entire radial peripapillary capillary density, inside disc VD, and sectoral peripapillary VD were automatically measured. On the retina, 6 × 6-mm scanning patterns were used for en face angiographic images. A full-thickness retinal scan was segmented into superficial capillary plexus (SCP) and deep capillary plexus (DCP). The SCP extended from 3 μm below the internal limiting membrane to 15 μm below the inner plexiform layer, while the DCP extended from 15 to 70 μm below the inner plexiform layer [14]. Each plexus was divided into foveal (diameter of 1 mm), parafoveal (diameter of 3 mm), and perifoveal (diameter of 6 mm) regions, and subanalysis of each area was performed (Fig 1). OCTA measurement was conducted by an experienced technician. While taking OCTA measurements, patients were instructed to look forward as much as possible for focusing on the primary target. Severe motion artifacts due to poor cooperation on the OCTA measurement, scan quality <6/10, or the presence of significant artifacts were excluded from the analysis.

Data were analyzed using SPSS statistical software, version 22.0 (IBM Corp., Armonk, NY, USA). Statistical significance was set at P<0.05. Intraocular VD of the paralysis eye was compared with that of the non-paralysis eye using the paired t-test. Changes in optic disc and macular vessel densities from diagnosis to recovery were also compared using the paired t-test. Multivariate linear regression analysis was performed to identify the clinical factors affecting intraocular vascularity, including initial angle of deviation, severity of duction limitation, and duration of symptom recovery.

## Results

Clinical demographics of the included patients are listed in Table 1. A total of 31 patients (21 males and 10 females) were enrolled in the study. Mean age of patients was 64.1±13.0 years (range: 31–84), and common underlying diseases were hypertension (n = 17, 54.8%) and diabetes (n = 12, 38.7%). The sixth nerve was most commonly affected nerve (n = 17, 54.8%), and the mean period until recovery was 3.0±1.6 months (range: 1–6). Maximum eyeball deviation at diagnosis was at an angle of 17.7±11.3 PD (range: 3–50) with duction limitation of grade -2.0±1.0 (range: -1 to -4).

**Table 1:** Clinical demographics of patients with vascular paralytic strabismus.

Variable	Value	Range
Number of patient (n)	31	-
Age at diagnosis (years)	64.1±13.0	31–84
Sex (M: F)	21 (67.7%):10 (32.3%)	-
Laterality (OD: OS)	17 (54.8%):14 (45.2%)	-
Duration on symptom onset (days)	8.6±7.6	1–21
Duration on symptom recovery (months)	3.0±1.6	1–6
Duration of follow up period (months)	4.6±2.3	2–12
Angle of deviation in paralytic eye (PD)	17.7±11.3	3–50
Maximal duction limitation (grade)	-2.0±1.0	-1–-4
Type of paralytic cranial nerve (n)		
3^rd^ nerve palsy	7 (22.6%)	-
4^th^ nerve palsy	5 (16.1%)	-
6^th^ nerve palsy	17 (54.8%)	-
Multiple	2 (6.5%)	-
Presence of underlying disease (n)		
Diabetes	12 (38.7%)	-
Hypertension	17 (54.8%)	-
Dyslipidemia	9 (29.0%)	-
Cardiovascular disease	4 (12.9%)	-
Cerebrovascular disease	10 (32.2%)	-

Values are presented as mean ± standard deviation or number (%)

M: F, male: Female; OD: OS, oculus dexter: Oculus sinister; PD, prism diopters

Table 2 shows a comparison of macular and peripapillary VD between the paralysis and non-paralysis eyes at diagnosis. First, there were no significant differences in the basic parameters of visual acuity, intraocular pressure, and refractive errors between the two eyes. Foveal VD of the SCP in the paralysis eyes (17.40±7.49%) was significantly lower than that in the non-paralysis eyes (19.45±6.40%; P = 0.034). Otherwise, there was no difference in the VDs of the DCP and optic disc vessels. Table 2 also shows the VD between the two eyes after the recovery of vascular paralytic strabismus. There was no significant difference in foveal VD of the SCP between the two eyes after recovery (P = 0.582), which was significantly different at the time of diagnosis; otherwise, there was no difference in other vascular structures.

**Table 2:** Comparison of macular and peripapillary vessel densities between paralysis eye and non-paralysis eye at diagnosis.

Parameter		Paralytic eye	Non-paralytic eye	P values
Visual acuity (LogMAR)		0.08±0.09	0.09±0.10	0.507
IOP (mmHg)		13.11±2.96	12.98±2.95	0.637
MRSE (D)		-0.37±1.91	-0.40±1.68	0.891
Macular vessel density at diagnosis (% area)				
SCP	Average	44.49±5.08	44.53±5.94	0.981
	Fovea	17.40±7.49	19.45±6.40	0.034*
	Parafovea	47.61±5.54	47.60±5.08	0.995
	Perifovea	46.34±4.63	46.44±4.29	0.906
DCP	Average	46.73±4.46	46.93±4.23	0.808
	Fovea	31.30±8.11	32.90±7.41	0.150
	Parafovea	50.15±5.01	51.06±3.13	0.390
	Perifovea	44.81±5.61	44.69±6.53	0.925
RPC density at diagnosis (% area)				
Whole image		45.55±3.07	48.03±3.15	0.251
Inside disc		45.55±5.89	46.83±4.99	0.205
Peripapillary	Average	50.42±4.04	50.84±3.74	0.403
	Superior	51.73±4.94	52.58±4.22	0.255
	Inferior	52.54±4.86	53.38±4.39	0.374
	Nasal	45.81±5.75	45.69±4.52	0.892
	Temporal	53.42±3.89	53.19±4.32	0.760
Macular vessel density at recovery (% area)				
SCP	Average	44.46±5.76	45.17±4.52	0.610
	Fovea	18.51±6.07	19.47±6.50	0.582
	Parafovea	47.89±4.18	48.82±5.32	0.340
	Perifovea	47.56±3.44	47.85±4.45	0.644
DCP	Average	46.92±3.38	47.24±4.35	0.621
	Fovea	32.31±6.80	33.32±6.10	0.329
	Parafovea	50.16±3.79	50.06±4.84	0.924
	Perifovea	44.63±6.07	44.88±5.71	0.607
RPC density at recovery (% area)				
Whole image		48.00±3.28	48.33±2.01	0.408
Inside disc		45.73±6.27	46.50±4.79	0.569
Peripapillary	Average	50.78±5.17	51.33±2.79	0.435
	Superior	52.67±4.68	52.93±4.13	0.702
	Inferior	53.30±5.11	53.78±4.54	0.556
	Nasal	46.26±6.06	45.93±4.08	0.649
	Temporal	54.19±3.69	53.89±3.07	0.638

The values are expressed as mean ± standard deviation.

Value with asterisk represents the associations that are statistically significant.

LogMAR = logarithm of the minimum angle of resolution; IOP, intraocular pressure; MRSE, mean refractive spherical equivalent; D, diopter; SCP, superficial capillary plexus; DCP, deep capillary plexus; RPC, radial peripapillary capillary

When analyzing the VD in the optic disc area, RPC whole image mainly (45.55±3.07%) and inside disc (45.55±5.89%) in paralysis eyes were numerically lowered than those in the non-paralysis eyes (RPC whole images 48.03±3.15%, inside disc 46.83±4.99%), even statistically non-significant. These values in paralysis eyes increased to the level of the non-paralytic eye after recovery (RPC whole image 48.00±3.28% versus 48.33±2.01%, inside disc 45.73±6.27% versus 46.50±4.79%).

**Fig 2:**
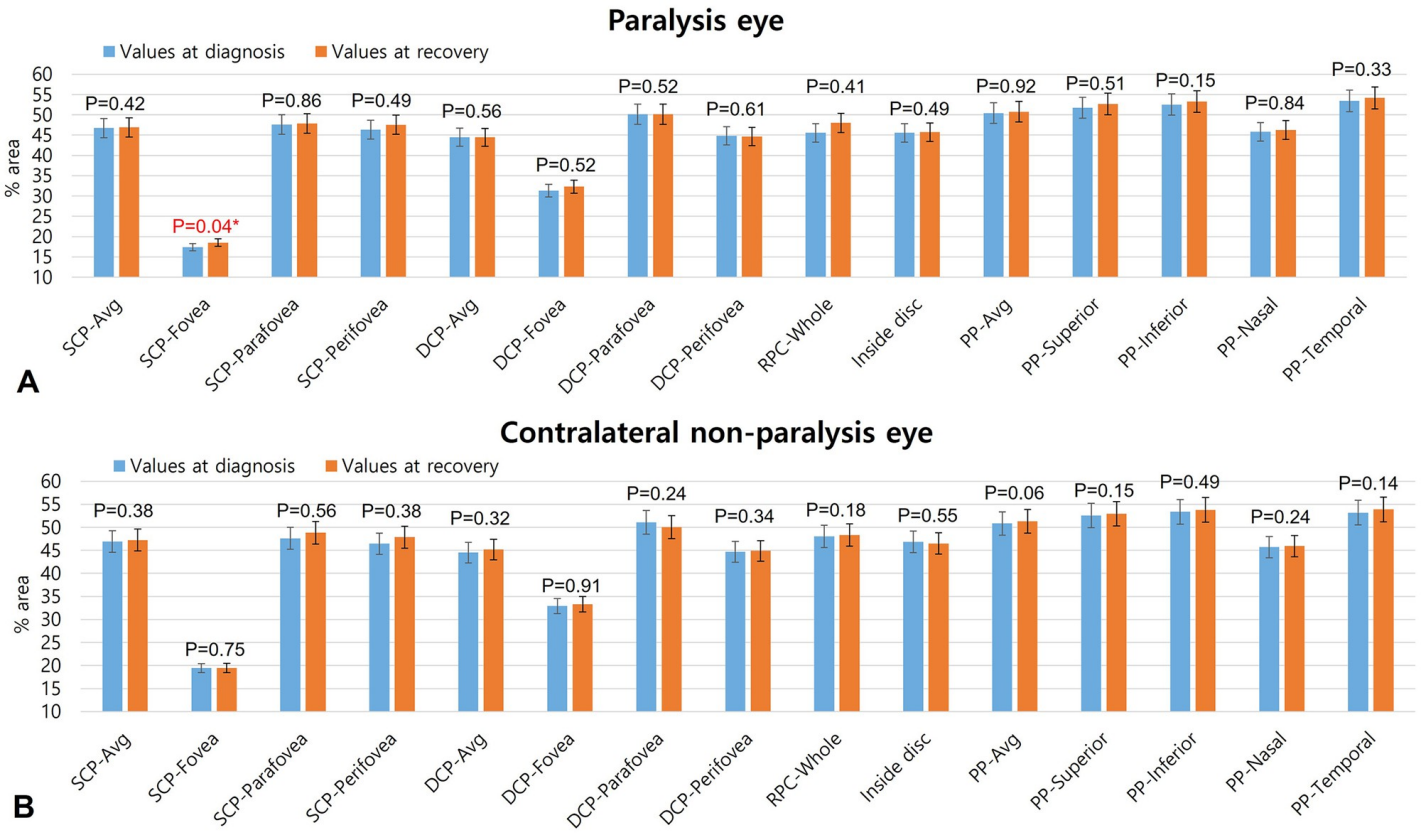
Changes in macular and peripapillary vessel densities between diagnosis and recovery. (A) shows values in paralysis eyes. (B) shows values in contralateral non-paralysis eyes. SCP, superficial capillary plexus; Avg, average; DCP, deep capillary plexus; RPC, radial peripapillary capillary; PP, peripapillary.

Fig 2 shows the changes in macular and peripapillary VDs in each eye between the diagnosis and recovery points. In paralysis eyes, foveal VD in SCP (17.40±7.49%) at diagnosis significantly increased (to 18.51±6.07%) after the strabismus recovered (P = 0.04). In other areas, there was no change in VD (Fig 2A). In the contralateral non-paralysis eyes, there was no change in VD in any values in the macula and optic disc area during the recovery periods of paralytic strabismus (Fig 2B).

Table 3 describes the effect of clinical factors on the optic nerve head and macular VD measurements at diagnosis in paralysis eyes. The foveal VD in the SCP showed a statistically significant association with the deviation angle (coefficient = -0.22, P = 0.04) and the grade of duction limitation (coefficient = 0.50, P = 0.01) at diagnosis. This means that the greater the maximum deviation angle and the more severe the eye movement restriction, the lower the foveal VD in the SCP. Otherwise, there was no significant association between anatomical vascular densities and clinical factors of paralytic strabismus.

**Table 3:** Multiple linear regression analysis of clinical factors on optic nerve head and macular vessel density measurements in paralysis eye (n = 31).

Vessel density			IOP (mmHg)	MRSE (D)	Angle of deviation (PD)	Duction limitation (Grade)	Duration on symptom recovery (months)
SCP	Average		0.02(0.93)	0.19(0.32)	-0.20(0.29)	0.21(0.28)	0.18(0.36)
	Fovea		0.13(0.51)	-0.18(0.35)	-0.22(0.04)*	0.50(0.01)*	-0.18(0.35)
	Parafovea		0.11(0.55)	0.09(0.66)	-0.29(0.29)	0.22(0.25)	0.03(0.88)
	Perifovea		-0.04(0.88)	0.23(0.22)	-0.25(0.18)	0.27(0.16)	0.20(0.29)
DCP	Average		0.18(0.34)	0.09(0.63)	0.20(0.29)	-0.18(0.34)	-0.05(0.81)
	Fovea		0.04(0.85)	-0.07(0.71)	-0.04(0.84)	0.34(0.06)	-0.16(0.41)
	Parafovea		0.39(0.03)	-0.01(0.94)	0.14(0.47)	-0.26(0.16)	-0.05(0.78)
	Perifovea		0.15(0.42)	0.10(0.61)	0.23(0.22)	-0.18(0.35)	-0.02(0.91)
RPC	Whole		-0.13(0.54)	0.16(0.41)	0.16(0.43)	-0.18(0.37)	0.17(0.40)
	Inside disc		0.09(0.66)	0.003(0.99)	0.20(0.34)	0.03(0.86)	0.03(0.87)
	Peripapillary	Average	-0.09(0.63)	0.09(0.68)	0.01(0.95)	-0.13(0.53)	0.04(0.85)
		Superior	-0.14(0.48)	0.00(0.99)	0.20(0.33)	-0.20(0.34)	-0.07(0.75)
		Inferior	-0.10(0.62)	-0.04(0.84)	0.00(0.99)	0.00(0.99)	-0.07(0.75)
		Nasal	-0.10(0.64)	0.33(0.11)	-0.06(0.79)	-0.26(0.19)	0.12(0.56)
		Temporal	0.09(0.64)	-0.18(0.37)	-0.10(0.62)	0.16(0.43)	0.13(0.53)

Number in the cell represents coefficient value with the P-value in parenthesis.

Value with asterisk represents the associations that are statistically significant.

MRSE, mean refractive spherical equivalent; D, diopter; PD, prism diopter; SCP, superficial capillary plexus; DCP, deep capillary plexus; RPC, radial peripapillary capillary

## Discussion

Vascular comorbidities such as diabetes, hypertension, dyslipidemia, and cerebral disease may affect intraretinal microvascular changes [15, 16]. Because vascular paralytic strabismus is known to occur due to ischemia of the cranial nerve, which innervates eye movement, this study was conducted assuming that vascular paralytic strabismus has the potential to affect the intraocular microvasculature, similar to other vascular comorbidities.

In neuro-ophthalmology, OCTA was initially applied to glaucomatous optic neuropathy [7, 17] or ischemic optic neuropathy to analyze microvascular abnormalities. Since then, it has been extended to diseases such as Leber hereditary optic neuropathy and optic neuritis [18, 19] and has now been applied to vascular diseases of brain origin, such as moyamoya vasculopathy and multiple sclerosis [20, 21]. To the best of the author’s knowledge, there have been no previous study on intraocular microvasculature analysis using OCTA in vascular paralytic strabismus.

The present study investigated changes in macular and peripapillary vascularity between disease development and recovery in patients with vascular paralytic strabismus using OCTA measurements. In an attempt to minimize the confounding bias toward the severity of systemic vascular risk factors affecting VD in OCTA [15], this study was designed to compare the paired eye, paralysis eye, and companion eye in each patient rather than comparing with the eyes of control subjects.

First, the overall VD values in the present study were lower than those reported in previous studies describing normal eyes. The value of VD as a control in previous studies using the same OCTA device as that used in the present study varies [15, 22, 23]; 48.96%-54.2% in macular SCP, 53.53%-59.3% in macular DCP, 48.58%-54.8% in whole-disc image VD, and 50.52%-62.7% in peripapillary VD. Although it is difficult to simply compare the value of VD between several studies and this study, because the age of the enrolled patients and their systemic conditions are different, the lower values in this study may be due to the enrolled patients’ systemic risk factors or the paralytic strabismus itself.

Comparing the VD of the paired eye in each patient, there was no significant difference in the VD of the optic disc area between the paralysis and contralateral non-paralysis eyes at the diagnostic point. In the macula, only foveal VD in the SCP was lower in the paralysis eye, and these values increased to the level of that in the contralateral eye after paralytic strabismus was recovered. Studies on the hemodynamic relationship with paralytic strabismus are rare [24, 25]. However, the results of the present study are similar to those of Shin et al. [24], who reported that transient thalamic blood flow reduction was evident on brain imaging in patients with ophthalmoplegic migraine. In their study, ipsilateral regional brain ischemia with ophthalmoplegia was observed, and the lesion reverted to normal during the symptom-free period. They concluded that reversible ischemia in the territories of the perforating cerebral artery may accompany ophthalmoplegia and possibly have some relationship with the clinical features. Accordingly, as suggested by Shin et al., transiently decreased VD in the SCP of the paralysis eye can represent microvascular ischemia on the ipsilateral side of the vascular paralytic strabismus.

The reason why the transient vascular changes occurred only in the SCP is unclear. Zhai et al. [26] reported that macular perfusion densities of the SCP in 3 × 3-mm scans and DCP in 6 × 6 -mm scans were lower in the deviating eyes than in the fixating eyes of patients with exotropia, which may be related to the constant lack of normal visual information received by the deviating eye. Unlike Zhai’s study, there was a significant perfusion decrease only in the SCP in this study. Although long-lasting misalignment visual stimulation exists in exotropia and there were no cases of acute onset paralytic strabismus, deviation itself may cause macular perfusion density in SCP changes in the deviating eye. Anatomically, DCP is vulnerable to ischemia or hypoxic injury owing to the presence of terminal anastomotic capillary networks [27, 28], and it is unknown why macular perfusion density decrease was observed only in SCPs in this study, and further studies with larger sample sizes are needed.

Another result of this study was a significant negative correlation between VD in the SCP and the deviation angle and a positive correlation between VD in the SCP and grade of duction limitation among clinical signs of vascular paralytic strabismus. These findings suggest that the more severe the paralytic strabismus, the more intraocular blood vessel ischemia. Systemic ischemic risk factors may cause cranial nerve malfunction and have an effect on intraocular ischemia, suggesting that the degree of paralytic strabismus due to cranial nerve palsy and the degree of intraocular ischemia were statistically correlated with each other. Another possible reason for the correlation between eye movement restriction and VD decrease may be the process of OCTA measurement. When patients underwent OCTA measurement of the paralysis eye, they were instructed to fix the target at the primary position through the paralysis eye. Focusing on the primary target with deviated and movement-restricted eyes may affect the intraocular vasculature transiently, and the degree of influence on VD would correspond to the severity of paralysis. In previous studies, horizontal eye movements causing strain on the optic nerve were described [29, 30]; however, they focused on optic disc tissue displacement and only evaluated tissue thickness or morphological changes using OCT measurements. Although the research remains controversial about the ocular morphologic changes that are induced by the eye movement, strain on macula and consequent macular VD change causing from adduction or abduction in paralysis eye can be suggested as a hypothesis in this result.

Time to recovery, which is another important clinical factor in paralytic strabismus, was not correlated with VD in this study. Ho et al. [5] reported that the presence of systemic vascular comorbidities was not associated with recovery from paralytic strabismus. Combining the findings of their research with those of this study, the risk factors for systemic ischemia or intraocular ischemia might not be directly related to the recovery time from paralytic strabismus. It is also thought that various factors, such as extraocular muscle tension itself and vascular ischemia, may influence recovery from the disease.

This study has several limitations. The sample size was small, and the number of cranial nerve subtypes was imbalanced; thus, the author could not conduct a sub-analysis according to strabismus type or paralysis severity. And, in comparison of VD values with previous studies, age-matched normative data with existing studies could not perform. VD may change with age but this study has a limitation that sub-analysis according to age could not perform. Instead, this study was a designed to compare paralysis and contralateral non-paralysis eyes in one patient to eliminate confounding factors regarding age as much as possible. Regarding the limitation of the retrospective design, baseline VD values of the enrolled patients before the development of paralytic strabismus could not be evaluated. If the baseline value before the disease onset had been identified, then the effect of paralytic strabismus on intraocular blood flow change could have been more clearly confirmed. In addition, choriocapillaris blood flow was also an important factor in demonstrating intraocular hemodynamics; however, an analysis of these parameters was not performed in this study. Further studies with large sample sizes, including choriocapillaris blood flow analysis, would be meaningful, and intraocular hemodynamic changes in cases of unrecovered cranial nerve palsy are also worth investigating. Rao et al. [15] reported that sex and the presence of underlying diseases affect VD in the OCTA of normal eyes, but there is a limitation in not controlling for the existence of various underlying vascular risk factors in enrolled patients. And not being able to analyze the duration or severity of a patient’s systemic diseases that may affect the VD values is another limitation. Although vascular cause was the most common etiology of acquired cranial nerve palsy [1, 13], this disease has various causes and processes. Another limitation of this study is that it analyzed only cases with vascular causes and complete recovery. OCTA analysis, including various etiologies or subtype analysis according to recovery, is worth studying.

Nevertheless, this study is meaningful in that it attempted to perform an intraocular structural analysis rather than a typical epidemiologic analysis of vascular paralytic strabismus. In conclusion, transient retinal ischemia of the paralysis eye was observed in a patient with paralytic strabismus, which corresponded to the degree of deviation angle and ocular motor restriction. These findings suggest that ischemia, which is the etiology of vascular paralytic strabismus, also affects intraocular blood flow. These findings are not yet sufficient to recommend the use of OCTA during the regular follow-up of vascular paralytic strabismus. However, OCTA measurement can be a tool for intraocular structural assessment and pathophysiological understanding in patients with paralytic strabismus.

## Supporting information

S1 Dataset (JPG)

S2 Dataset (JPG)
