# Supplementary figures and images for: Intraocular vascular analysis using optical coherence tomography angiography in patients with vascular paralytic strabismus
Source: PLoS One. 2022 Sep 13;17(9):e0272524. doi: 10.1371/journal.pone.0272524 (PMC9469969; doi:10.1371/journal.pone.0272524)

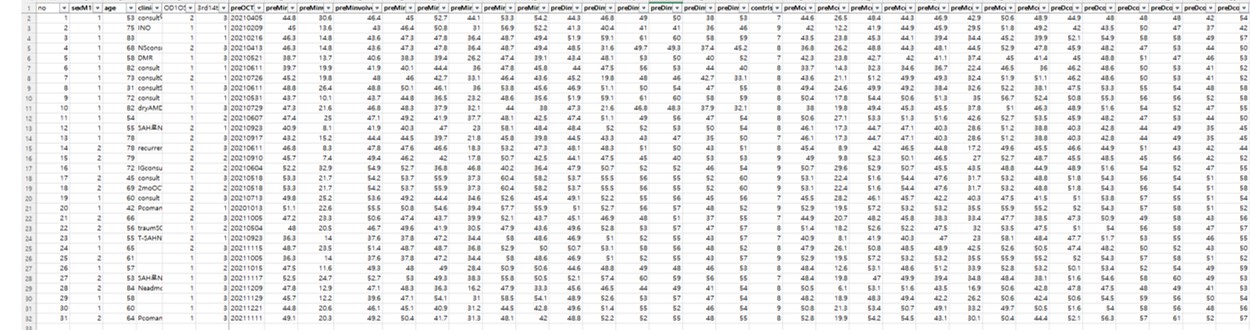

Supplement: S1 Dataset — (JPG) [file pone.0272524.s001.jpg]

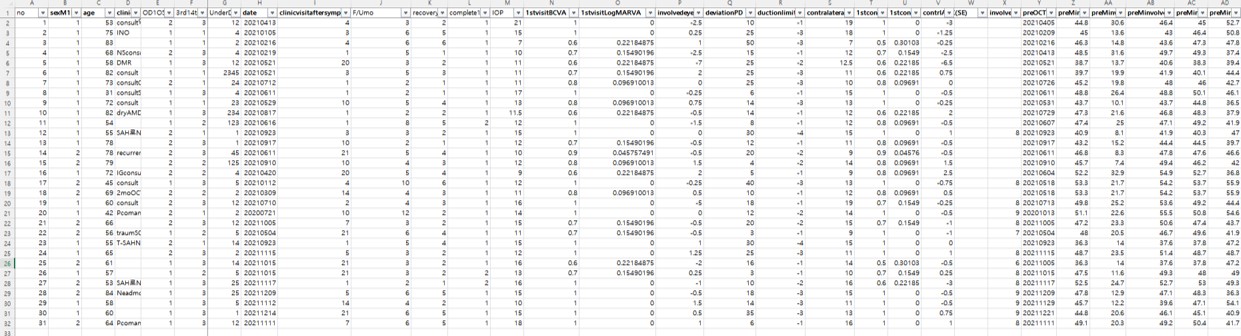

Supplement: S2 Dataset — (JPG) [file pone.0272524.s002.jpg]
